# Intracorneal implantation of a misdirected foldable intraocular lens during phacoemulsification surgery: a case report

**DOI:** 10.5935/0004-2749.2021-0093

**Published:** 2022-09-06

**Authors:** Mustafa Ilker Toker, Erman Bozali, Mustafa Unal, Ayse Vural Ozec, Haydar Erdogan

**Affiliations:** 1 Ulucanlar Eye Training and Research Hospital, University of Health Sciences, Ankara, Turkey; 2 Department of Ophthalmology, School of Medicine, Sivas Cumhuriyet University, Sivas, Turkey; 3 Department of Ophthalmology, School of Medicine, Akdeniz University, Antalya, Turkey

**Keywords:** Lens implantation, intraocular, Lenses, intraocular, Phacoemulsification, Wound healing, Cataract, Visual acuity, Implante de lente intraocular, Lentes intraocular, Facoemulsificação, Cicatrização, Catarata, Acuidade visual

## Abstract

A 59-year-old man presented with a unilateral blurring of vision in his left eye. His left eye’s visual acuity was hand movements level. He underwent phacoemulsification surgery, and an intrastromal posterior chamber intraocular lens was implanted. The intrastromal intraocular lens was extracted and a new intraocular lens was implanted. Usinge the Snellen chart, the final best-corrected visual acuity was 20/40. With this case report, we wish to emphasize that a single stepwise clear corneal incision merged with wound-assisted intraocular lens injections can result in intraocular lens misdirection into the corneal stroma. As a result, while performing a misdirected intraocular lens removal, we recommend that the wound be carefully constructed.

## Abstract (pt)

Um homem de 59 anos apresentou embaçamento visual unilateral no olho esquerdo. Sua acuidade visual nesse olho era no nível de movimentos da mão. O paciente havia se submetido a uma cirurgia de facoemulsificação em que foi feita a implantação intraestromal de uma lente intraocular de câmara posterior. Foi feita a extração dessa lente intraestromal intraocular e uma nova lente intraocular foi implantada. A melhor acuidade visual corrigida final foi de 20/40 pela tabela de Snellen. Com este relato de caso, os autores desejam apontar que uma incisão de degrau único em córnea clara, quando combinada com a injeção de uma lente ocular através da incisão, pode levar a um direcionamento incorreto da lente intraocular para dentro do estroma corneano. Portanto, recomenda-se uma construção cuidadosa da incisão ao se remover uma lente intraocular direcionada incorretamente.

## INTRODUCTION

A cataract is one of the most common health problems causing visual impairment worldwide. Despite the widespread use of phacoemulsification in developed countries, there are approximately 20 million blind individuals currently^(1)^. Surgical standards for phacoemul-sification surgery have excelled using refined surgical techniques and surgical equipment, resulting in reduced complication rates^(2,3)^. The performing surgeon’s spatial awareness and precise hand maneuvers determine surgical success^(4)^. Using the wound-assisted technique, surgeons can implant the intraocular lens (IOL) through a smaller incision^(5)^. The IOL is inserted by positioning the tip of the cartridge against the incision rather than into the anterior chamber^(5)^. The current case report demonstrates a misled intrastromal IOL implantation following routine phacoemulsification surgery utilizing the wound-assisted technique.

## CASE REPORT

**Figure 1:**
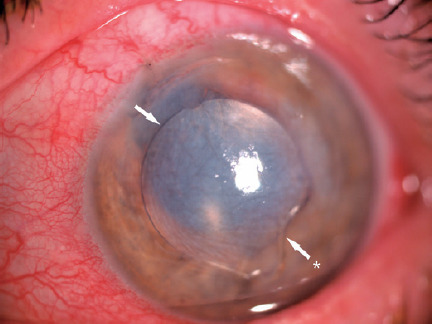
Intraocular lens misplaced into the corneal stroma. (White arrow indicates the margin of the IOL optic; white arrow with an asterisk indicates the inferior haptic of the IOL).

After cataract surgery on his left eye in 2012, a 59-year-old man was referred to a tertiary referral hospital, Cumhuriyet University School of Medicine Department of Ophthalmology outpatient clinic, with a unilateral blurring of vision. He had undergone surgery by an experienced ophthalmologist in a state hospital. The ophthal-mological findings included decreased best-corrected visual acuity of the left eye to hand movements. Slit-lamp biomicroscopic examination revealed that the IOL had completely penetrated the corneal stroma (Figure 1). Under topical anesthesia, a 2.4 mm superonasal clean corneal incision was created and an uneventful cataract extraction by phacoemulsification was performed. The single-piece foldable hydrophobic acrylic IOL was implanted using an injector cartridge system manufactured by RET Inc., Korea (Zaraccom F260 posterior chamber IOL). Late in the procedure, the surgeon realized that the IOL had progressed and was inserted intrastromal. Following suturing the main corneal incision with a 10/0 nylon suture, the patient was sent to our clinic.

**Figure 2:**
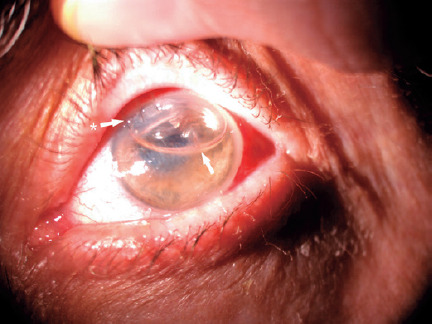
First postoperative day after the removal of the intrastromal intraocular lens. (White arrow indicates the air bubble in the anterior chamber; white arrow with an asterisk indicates the limbal sutures).

**Figure 3:**
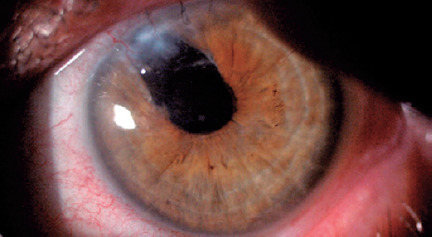
Aphakic eye of the patient before secondary intraocular lens implantation.

**Figure 4:**
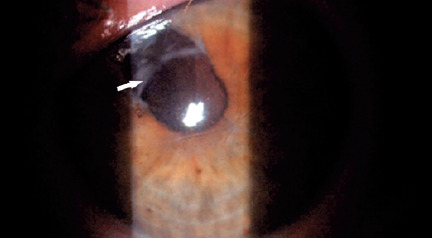
After implantation of the secondary intraocular lens in the capsular bag. (White arrow indicates the margin of the IOL optic placed in the capsular bag).

An experienced ophthalmologist removed the IOL the next day after increasing the size of the corneal wound to 6 mms. The anterior chamber was next injected with an air bubble, and the widened incision was sutured with 10/0 nylon (Figure 2). The corneal edema began to resolve on the second postoperative day, and the best-corrected visual acuity was counting fingers at 2 feet. To minimize corneal scarring, topical dexame-thasone drops were started and administered 4 times per day for 3 months. Secondary IOL implantation was scheduled one year after the IOL explantation (Figure 3). The ultimate best-corrected visual acuity with the Snellen chart was 20/40 after the secondary IOL was implanted into the capsular bag (Figure 4). However, since the patient was lost to follow-up, we were unable to undertake postoperative corneal exams such as corneal topography or anterior segment optical coherence tomography to illustrate the healing process and the ultimate state of the cornea.

## DİSCUSSİON

IOL implantation using intraocular lens injectors is simple, and ophthalmic surgeons often require just a short period to overcome the learning curve^(6)^. Several complications have previously been reported, including the capture of the haptics inside the cartridge, injector related marks on the optics of the IOL, broken haptics, the lens unfolding upside down in the anterior chamber, the haptics not being released from the optics, and the cracking of the IOL and the cartridges^(6,7,8)^.

In 2008, Hogden et al. reported a case in which the IOL was inserted into the corneal stroma approximately 4 mms from the limbus, sparing the visual axis^(9)^. With the evolving techniques, smaller incisions can lead to difficulties during the insertion of the IOLs. The wound-assisted injection has lately become popular among surgeons all over the world. Single stepped clear corneal incisions merged with wound-assisted IOL injections may result in IOL misdirection into the corneal stroma. Shiba et al. compared push and mechanical injectors and reported that with the push injector, the danger of quick and aggressive IOL injection may arise due to lower resistance to insertion when the IOL is released from the cartridge^(10)^. A mechanical injector, on the other hand, may prevent the potential of unexpected ejection owing to consistent pressure on the plunger. The vital key points of IOL injection are attentive wound construction and observing the IOL as it passes through the wound without any corneal injury. The injector tip should be beveled down into the anterior chamber. In this case, injection of the foldable IOL with a push injector into the corneal stroma is likely owing to the tip of the injector being in touch with the wall of the corneal stroma rather than being directly in the anterior chamber. Furthermore, we hypothesize that the surgeon may have encountered unexpected resistance while implanting the IOL, although he presumably continued injecting the IOL intrastromal. Rapid injection of the IOL with excessive plunger pressure may potentially result in IOL misplacement into the cornea. To the best of our knowledge, this is the first case in the literature reporting complete intrastromal implantation of an acrylic IOL.

As a complication of wound-assisted IOL implantation, an IOL can be implanted intracorneal. In this situation, the IOL must be removed gently without causing any additional damage to the cornea by enlarging the entrance port. When explanting the IOL, the wound should be constructed in such a way that it does not cause any additional damage to corneal stroma by enlarging the incision on the anterior stromal side adjacent to the anterior surface of the IOL. In addition, we anticipated that injecting air into the anterior chamber after the lens was removed would aid in the healing process of the corneal stroma.

This kind of complication may also occur in individuals whose corneal endothelium is loosely attached to the stroma. In such circumstances, IOL injection should be interrupted promptly and a careful inspection should be performed to determine the likely cause. When adopting the wound-assisted approach, ophthalmologists who commonly conduct phacoemulsification surgery should keep the likelihood of corneal intrastromal implantation of the IOL in mind.
